# Editorial: Methods and applications in metabolic physiology

**DOI:** 10.3389/fphys.2023.1156826

**Published:** 2023-02-09

**Authors:** Amy C. Arnold, Kamal Rahmouni

**Affiliations:** ^1^ Department of Neural and Behavioral Sciences, Pennsylvania State University College of Medicine, Hershey, PA, United States; ^2^ Department of Neuroscience and Pharmacology, University of Iowa Carver College of Medicine, Iowa City, IA, United States

**Keywords:** metabolic, metabolomics, biomarkers, energy balance, rodent models, clinical

Metabolic physiology has remained a mainstay of research for over a century, as metabolism and metabolites are paramount to the biological processes that provide energy to maintain homeostasis. This area of research has become particularly important given the recent global surge of chronic diseases associated with dysregulated metabolism such as obesity, diabetes, non-alcoholic fatty liver disease, and cardiovascular disease. The goal of this Research Topic, “*Methods and applications in metabolic physiology*,” is to highlight innovative methods and emerging technologies to investigate metabolic processes that maintain physiological homeostasis, and how these processes become disturbed during disease pathophysiology at the molecular, cellular, organ, and organism levels.

As the prevalence of obesity and related metabolic and cardiovascular complications continues to rise globally, there is an urgent need for accurate and high-resolution methods to study integrated metabolic and cardiovascular physiology in animal models. The article by Reho et al. provides a technical guide for high-throughput and high-resolution comprehensive cardiometabolic phenotyping in mice and rats, with methods spanning measurement of body composition, electrolyte accumulation and flux, energy storage and flux, physical activity, ingestive behavior, ventilatory function, blood pressure, heart rate, autonomic function, and gut microbiota. Importantly, these technologies can be used for diverse purposes including to gain insight into normal physiological functioning, pathophysiology in the context of numerous diseases, and responses to dietary or pharmacological interventions. The article by Rial-Pensado et al. uses some of these metabolic phenotyping approaches to show that environmental temperature modulates the systemic and central actions of thyroid hormones on brown adipose thermogenesis and energy balance in rats in a complex manner. This finding may have implications for experimental design considerations as well as to improve understanding of the physiological role of thyroid hormones in adaptations to environmental temperature.

A rapidly growing research area in metabolic physiology relates to the evaluation of bioactive metabolites using omics approaches. In this regard, metabolomics allows for the identification and profiling of small molecule metabolites, providing a window into the molecular signature of cellular and organ level metabolic physiology (Wishart, 2019). This technique has also been used to advance clinical applications such as identifying biomarkers for diagnosis, treatment, and outcome prediction in diseases. Two articles in this Research Topic highlight the use of metabolomics to identify biomarkers for cardiovascular disease diagnosis and outcome prediction. Jiang et al. used untargeted and targeted metabolomics approaches to identify dysregulated metabolites in the sera of patients with coronary heart disease. They created a clinical model incorporating the dysregulated metabolite oxyneurine with triglycerides and body weight, which provided a robust and promising biomarker for early diagnosis of coronary heart disease. Liu et al. performed non-targeted metabonomics analysis of plasma samples to identify biomarkers to help stratify post-operative risk in patients with ST-segment elevation myocardial infarction (STEMI) undergoing percutaneous coronary intervention. They developed a robust multifactor early survival prediction model for in-hospital STEMI patients that integrated seven metabolites with six clinical indicators, which may help stratify risk in the early stage of morbidity and inform on administration of intensive care to high-risk groups.

In addition to omics approaches, next-generation sequencing technologies have emerged as a valuable strategy to evaluate genetic changes in the context of cellular metabolism and metabolic physiology. In particular, RNA-sequencing has been used as a powerful approach for transcriptome-wide analysis of differential gene expression in blood and tissues. More recent technologies, such as single-cell RNA-sequencing (scRNA-seq) have further expanded on these capabilities to allow analysis of the transcriptomes of individual cells (Hwang et al., 2021). This is particularly important to understand physiology and pathophysiology in metabolically-relevant tissues with heterogeneity in terms of specialized cell subtypes. Current methods for scRNA-seq, however, often require intact, live cells obtained from fresh tissue, thus providing challenges for use in clinical applications. The article by McRae et al. provides a novel workflow for cryopreservation of human intestinal mucosal biopsies and subsequent isolation with fluorescence-activated cell sorting coupled with scRNA-seq. This workflow was validated in samples from a small cohort of human subjects and cryopreserved tissue was also successfully used to generate short-term primary intestinal cultures as well as for *ex vivo* physiological assays. Overall, this protocol provides a more accessible approach for single-cell transcriptomics in intestinal mucosal biopsies, and may help to inform on changes in the intestinal environment in disease states. Additional studies are needed to determine if a similar protocol would provide useful to study single-cell transcriptomics in other tissue types with cellular heterogeneity.

In summary, a major challenge in the field of metabolic physiology has been to identify novel targets and pathways that regulate homeostasis, and how alterations in these pathways result in metabolic dysfunction and disease. Thus, novel approaches to study metabolic physiology have become critically important to improve our understanding of the pathophysiology of numerous metabolic-related disease states in both animal models and clinical populations and to develop appropriate novel therapeutic approaches. The articles highlighted in this Research Topic use a variety of sophisticated and cutting-edge technologies to advance the field of metabolic physiology, with a focus on whole animal phenotyping, omics, and next-generation sequencing approaches.

## References

[B1] Hwang B. Lee J. H. Bang D. (2021). Author Correction: Single-cell RNA sequencing technologies and bioinformatics pipelines. Exp. Mol. Med. 53 (5), 1005. 10.1038/s12276-021-00615-w 34045654 PMC8178331

[B2] Wishart D. S. (2019). Metabolomics for investigating physiological and pathophysiological processes. Physiol. Rev. 99 (4), 1819–1875. 10.1152/physrev.00035.2018 31434538

